# Network Pharmacology Analysis of the Identification of Phytochemicals and Therapeutic Mechanisms of *Paeoniae Radix Alba* for the Treatment of Asthma

**DOI:** 10.1155/2021/9659304

**Published:** 2021-09-14

**Authors:** Jingwei Wang, Ling Peng, Lu Jin, Huiying Fu, Qiyang Shou

**Affiliations:** ^1^Second Clinical Medical College, Zhejiang Chinese Medical University, China; ^2^Academy of Chinese Medical Science, Zhejiang Chinese Medical University, China; ^3^Zhejiang Provincial Key Laboratory of Sexual Function of Integrated Traditional Chinese and Western Medicine, Hangzhou, China; ^4^Department of Respiratory Disease, Zhejiang Provincial People's Hospital, Hangzhou, Zhejiang Province, China

## Abstract

**Background:**

*Paeoniae Radix Alba* (PRA), the root of the plant *Paeonia lactiflora* Pall., has been suggested to play an important role for the treatment of asthma. A biochemical understanding of the clinical effects of *Paeoniae Radix Alba* is needed. Here, we explore the phytochemicals and therapeutic mechanisms via a systematic and comprehensive network pharmacology analysis.

**Methods:**

Through TCMSP, PubChem, GeneCards database, and SwissTargetPrediction online tools, potential targets of active ingredients from PRA for the treatment of asthma were obtained. Cytoscape 3.7.2 was used to determine the target of active ingredients of PRA. Target protein interaction (PPI) network was constructed through the STRING database. The Gene Ontology (GO) biological process and Kyoto Encyclopedia of Genes and Genes (KEGG) pathway enrichment analysis were analyzed through the biological information annotation database (DAVID).

**Results:**

Our results indicate that PRA contains 21 candidate active ingredients with the potential to treat asthma. The enrichment analysis of GO and KEGG pathways found that the treatment of asthma by PRA may be related to the process of TNF (tumor necrosis factor) release, which can regulate and inhibit multiple signaling pathways such as ceramide signaling.

**Conclusions:**

Our work provides a phytochemical basis and therapeutic mechanisms of PRA for the treatment of asthma, which provides new insights on further research on PRA.

## 1. Introduction

Asthma is a common condition due to chronic inflammation of the lower respiratory tract [1]. Asthma exhibits recurrent episodes of wheeze, cough, chest tightness, and shortness of breath. It arises from heterogenic gene-environment interactions which are not fully understood. Airway hyperresponsiveness, reversible airflow obstruction, and bronchial hyperresponsiveness are characteristic features of asthma [2]. Most asthma is associated with sensitization of the airways to common allergens. Upon exposure to allergen, early response was the result of activation of airway mast cells in an IgE-dependent way with release of the rapidly acting granule-associated preformed mediators, as histamine and tryptase, to contract airway smooth muscle, promote vascular leakage, and stimulate mucus secretion. Allergic response such as interleukins- (IL-) 4, IL-5, IL-6, and TNF-*α*, in concert with the inflammatory mediators, stimulates the recruitment and activation of secondary effector cells, starting with neutrophils, followed by eosinophils and then T lymphocytes to cause late-phase airway narrowing and airway hyperresponsiveness [3]. This in turn produces long-term changes in the structure of the affected organs and substantial abnormalities in their function, which impact the quality of life.

Traditional Chinese medicine (TCM) has a long-lasting history of using herbal medicine in the treatment of various respiratory diseases [4]. There is increasing scientific evidence supporting the use of TCM for the treatment of asthma. Possible mechanisms include anti-inflammation, inhibition of airway smooth muscle contraction, and immunomodulation [5]. *Paeoniae Radix Alba* (Bai Shao, also known as Chinese peony), the root of the perennial herbaceous plant *Paeonia lactiflora* Pall. of the buttercup family, has the effects of nourishing blood and regulating menstruation, restraining yin and antiperspirant, softening liver and relieving pain, and suppressing liver-yang [6]. Our previous studies investigated the antiallergic role of total glucosides of peony, indicating that total glucosides of peony have an effect on the allergic reaction of mice [7]. Thus, we speculate that the total glucosides of peony should have a therapeutic effect on asthma. In this study, network pharmacology was utilized to analyse the active ingredients, drug targets, and key pathways of PRA for the treatment of asthma.

## 2. Methods

### 2.1. Composition of Chinese Medicine White Peony

TCMSP database was used to obtain the pharmaceutical ingredients with “*Paeoniae Radix Alba*” as the key word. The ingredients that meet the criteria with oral biological degree (OB) > 20% and drug − like properties (DL) > 0.18 at the same time were selected as the active ingredients of PRA. Effective active ingredients of PRA contained in the database were queried and screened.

### 2.2. Prediction of Small Molecule Target Protein

Based on the TCMSP and DrugBank databases, target proteins corresponding to small molecules were sorted out, while components without target proteins and duplicate targets were deleted. Using the UniProt (http://www.Uniprot.org/) database, screened targets are converted into gene names, full protein names are corresponded to the gene abbreviations, and the component target-gene data table is made.

### 2.3. Chemical-Gene Network Analysis

According to the chemical molecule-gene relationship obtained by the analysis, component target-gene data were imported into the Cytoscape 3.7.2 software. The data were analyzed and displayed graphically in the form of a network diagram. Different nodes are used to represent data types, and connections are used to represent interaction relationships. Topological analysis is performed, and the core target of PRA is selected according to the node degree. The node degree is one of the main data methods to determine the key nodes. The higher the node degree, the more important the role of the node in the network.

### 2.4. Asthma Gene Acquisition and Analysis

The UniProt database (https://www.uniprot.org/) and TTD data (https://db.idrblab.org/ttd/) database were searched for asthma-related targets. Duplicate targets were deleted, and information acquired were collected.

### 2.5. Gene Pathway and Function Analysis

The DAVID database (https://david.ncifcrf.gov/) and Metascape database (https://metascape.org/) were used to analyze the GO function and KEGG pathway enrichment analysis of the potential targets of PRA for the treatment of asthma. GOTERM_BP_DIRECT (biological process), GOTERM_CC_DIRECT (cell composition), and GOTERM_MF_DIRECT (molecular function) are saved in Gene_Ontology; KEGG_PATH-WAY was saved in Pathways.

## 3. Results

### 3.1. Screening of Active Ingredients of Paeonia

Oral administration is the most common route of traditional Chinese Medicine preparations. OB is an effective index to evaluate the clinical efficacy of traditional Chinese medicine. Through the TCMSP database, combined with “OB > 20%” and “DL > 0.18”, 21 active ingredients in PRA that can be orally absorbed were screened out, as shown in Table 1.

### 3.2. Target Prediction Results of Active Ingredients in White Peony

Reverse docking through the SwissTargetPrediction website was used using “*Homo sapiens*.” Target proteins of the active ingredients were screened, and gene target corresponding to each ingredient was acquired. A total of 147 targets after removing 90 targets were sort out, and the target-gene pairs were obtained, which are shown in Table 2.

### 3.3. Small Molecule Target Protein

**Figure 1:**
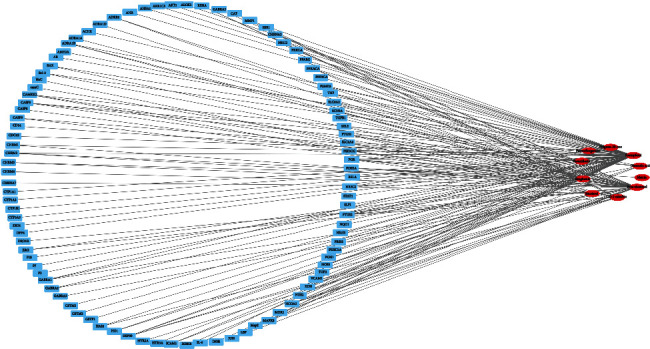
Part of main active ingredients of PRA.

Based on the TCMSP and DrugBank databases, the target protein relationship of each chemical small molecule after integration was obtained.  Figure 1 is a network diagram of the effective small chemical molecules and target proteins contained in PRA. The network diagram contains 42 nodes (10 small chemical molecules and 96 target proteins) and 145 edges.  Table 3 shows the degree table of the small chemical molecules and target proteins of PRA. In this network, kaempferol with the highest degree of connectivity regulates 63 target proteins, followed by beta-sitosterol, sitogluside, (+)-catechin, etc. The target protein with the highest degree of connectivity is PGR, which is regulated by 6 small chemical molecules, followed by NCOA2, HSP90, PTGS1, PTGS2, and so on.

### 3.4. Functional Analysis

**Figure 2:**
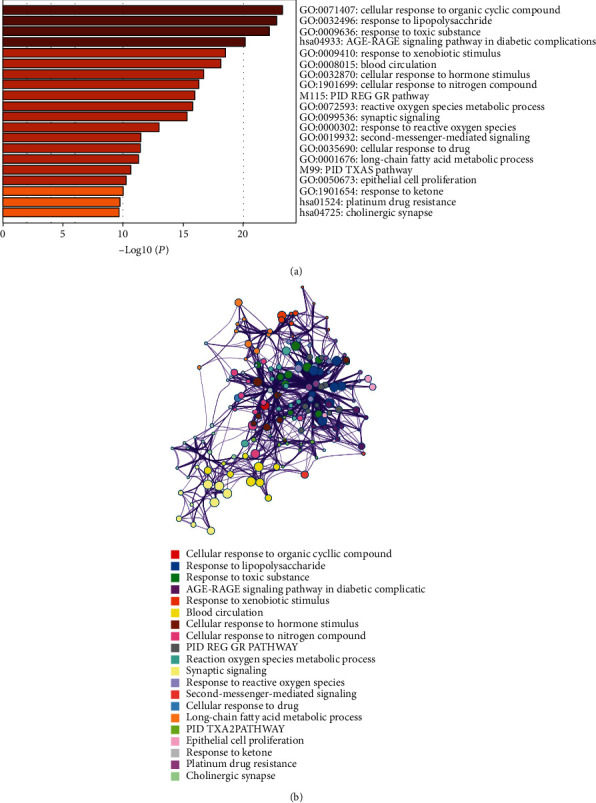
Graph of the GO function analysis bar and PPI. (a) GO analysis. (b) PPI network.

**Figure 3:**
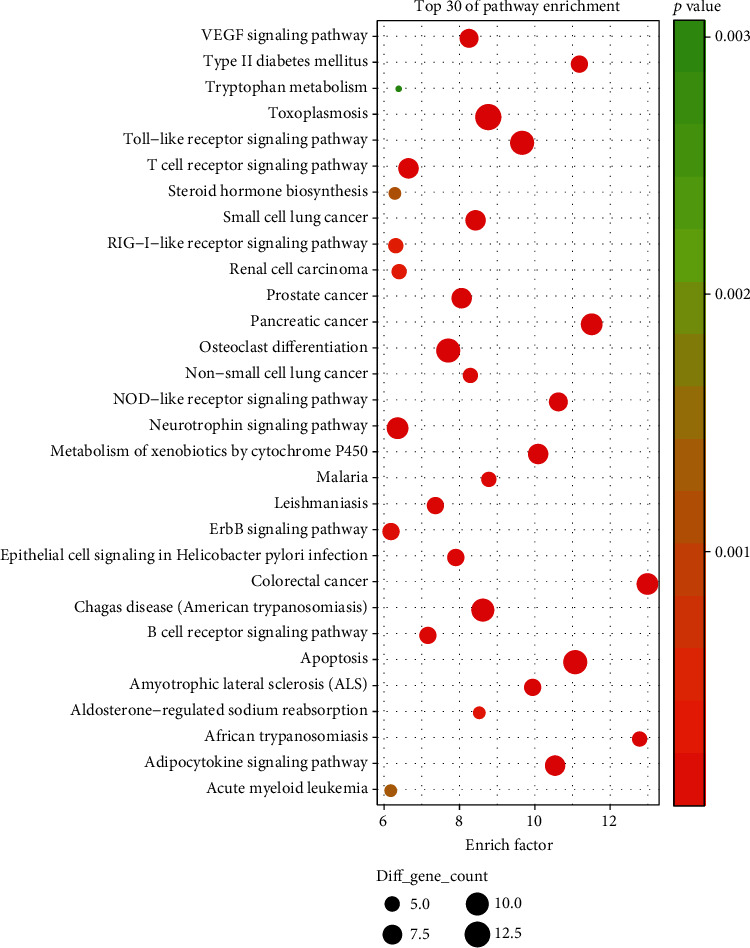
KEGG-enriched bubble chart.

**Figure 4:**
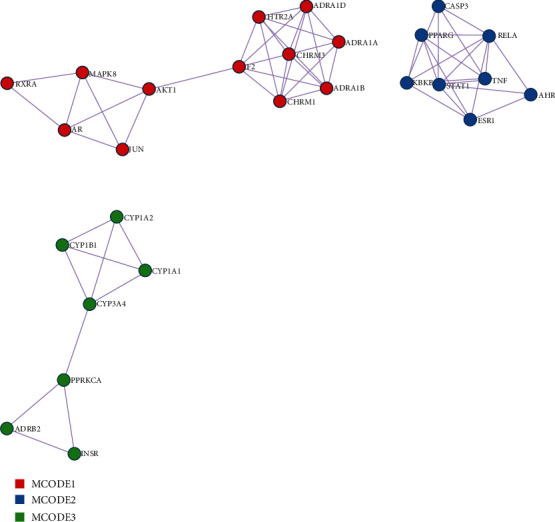
Protein interaction network of active ingredient in PRA.

Based on the DAVID tool, the GO function and KEGG pathway enrichment analysis of the target genes of PRA were performed, and the significance threshold was selected as the correction (Benjamini *P* value < 0.01).  Figure 2 is the GO analysis diagram. The most significant biological processes are cellular response to organic cyclic compound, response to lipopolysaccharide, and response to toxic substance.  Figure 3 shows the enrichment of the first 30 pathways. The most significant pathways are toxoplasmosis, Toll-like receptor signaling, osteoclast differentiation, apoptosis, and the Chagas disease. The interaction between related proteins can be classified into positive regulation of vasoconstriction, toxoplasmosis, and estrogen metabolic process, as shown in Figure 4, among which the most widely involved is the vasoconstriction.

### 3.5. Key Gene Screening of Asthma

**Figure 5:**
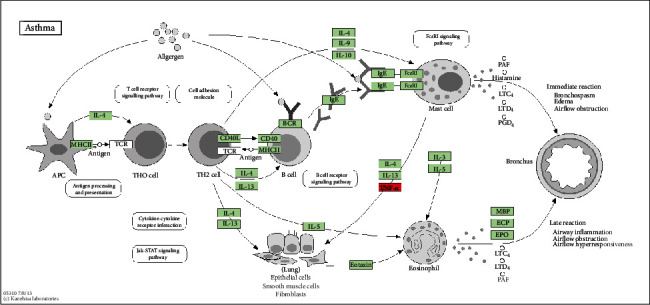
The pathway map of asthma.

We searched for disease targets in databases as Delegated Database Tree (DDT), DrugBank, and DisGeNET, and a total of 153 asthma-related targets were obtained. The intersection of these targeted genes with the previous targets of the active substance of PRA was analyzed. Finally, 13 genes are both target genes (Table 4). KEGG enrichment analysis was performed on these 13 key genes, and *P* < 0.05 was chosen as the threshold, which is shown in Table 5. Through the enrichment analysis of the coacting targets, the red node in Figure 5 indicates the key upregulated gene of asthma, which only contains *TNF*. We believe that only *TNF* is the key gene in the treatment of asthma with white peony drugs, and some downregulated genes such as IL-4 and IL-13 will affect the regulation of asthma.

## 4. Discussion

Asthma is a chronic disease characterized by inflammation hyperreactivity of the airways [8]. It is a complex syndrome with many clinical phenotypes in adults and children, which consists different phenotypes that share common features with distinct etiologies and pathophysiological pathways leading to disease [9]. The inhaled allergen encounters antigen presenting cells (APCs) in the airways. After recognizing the antigen and being activated by APCs, naïve T cells differentiate into Th2 cells, and the activated Th2 cells stimulate B cells to form IgE. IgE molecules then bind to IgE receptors on mast cells. The IgE crosslinking of allergens and mast cells will release biologically active mediators (histamine and leukotrienes) through degranulation, leading to direct symptoms of allergies [10]. Mast cells also release chemokines, which help the recruitment of inflammatory cells, especially eosinophils, which secret IL-5 to promote their proliferation and differentiation from bone marrow progenitor cells [11]. The activated eosinophils will release toxic particles and oxygen-free radicals, which leads to tissue damage and promotes the development of chronic inflammation.

The human TNF-*α* gene is about 2.76 kb in length and consists 4 exons and 3 introns. It is closely linked to the MHC gene group and is located on the 6^th^ and 17^th^ chromosomes, respectively. The natural form of TNF-*α* exerts its biological effects is a homotrimer. TNF-*α* is mainly produced and secreted by activated alveolar macrophages with a variety of biologically active cytokines, which can stimulate neutrophils to cause degranulation and an increase in respiratory burst activity. TNF-*α* can promote the adhesion of neutrophils to endothelial cells [12], and it can also promote the activation of monocytes and accelerate the expression of IL-2 receptors [13]. The phagocytic function of alveolar macrophages causes damage, which stimulates the activation of inflammatory mediators and induces the body to produce asthma symptoms. The asthma pathway map of our study indicates that TNF-*α* is the key target of PRA for the treatment of asthma. Among them, TNF-related active ingredients are kaempferol and paeoniflorin. Kaempferol suppresses eosinophil infiltration and airway inflammation in airway epithelial cells and in mice with allergic asthma through inhibition of TNF-*α* [14]. Kaempferol can inhibit ceramide signaling pathway, which reduce the release of TNF-*α*, reduce the transcription of MMP genes, and reduce the expression of MMP. Due to the reduced expression of MMP, it can effectively protect the extracellular membrane (ECM) and basement membrane in the lung from dissolution and avoid asthma symptoms [15]. Paeoniflorin attenuates adipocyte lipolysis and inhibits the phosphorylation of ERK, JNK, and IKK stimulated by TNF-*α* [16]. Thus, previous work on the compounds correlates with the results of our bioinformatic analysis of network pharmacology exploration of asthma and PRA.

Furthermore, there are many other genes which play roles in the coacting target genes, such as *CHRM*, *COX*, and *PTGS2.* Kaempferol has anti-inflammatory effects by inhibiting interleukin-4 (IL-4) kinase and downregulating the NF-*κ*B pathway [17]. By inhibiting IL-4 expression and cyclooxygenase 2 (COX2), kaempferol has a significant inhibitory effect on NADPH oxidase activity. When kaempferol reduces the reactive oxygen species (ROS) through the direct binding of NADPH oxidase, it can promote COX2 induction, thereby inhibiting epithelial thickening, indicating that this component can have a better adjuvant therapeutic effect on respiratory diseases through anti-inflammatory effects [18].

There are many highly sensitive cough receptors in the throat and trachea. When the mucosa of respiratory tract is stimulated by foreign bodies or secretions in the respiratory tract, it will produce a cough response through a series of neural reflexes, with the intention of expelling the secretions in the respiratory tract. A series of mechanical or chemical stimuli, such as the inhalation of harmful gases, the invasion of pathogenic microorganisms, the inflammatory factors produced by airway inflammation, and the contraction of airway smooth muscles, can all be used as inducements to cause the receptors to transmit signals to the cough center after the body is stimulated. Through network pharmacological analysis, it is found that PRA can act on key targets in cough response-related pathways. Beta-sitosterol and sitogluside, the active ingredients in PRA, can regulate the concentration of Ca^2+^ through the calcium ion pathway and inhibit the activation of CHRM2 and CHRM3 on the bronchial smooth muscle, thereby inhibiting the contraction of the bronchial smooth muscle and reducing cough [19]. The stimulus received by the receptors, in turn, plays a role in relieving cough. In addition, the Ca^2+^ produced by this reaction can directly bind to ion channels and can indirectly participate in the process of other signaling pathways related to the cough and asthma reaction [20].

Asthma is mainly related to allergic inflammation and neurological reactions. When the mucous membrane of the respiratory tract tissue is stimulated by allergens, it will promote the production of large amounts of IgE antibodies, triggering type I allergies. IgE high-affinity receptor “Fc*ε*RI” is expressed on the surface of a variety of immune cells (mast cells, B cells, eosinophils, etc.). The massively secreted IgE can trigger allergic inflammation cascades by activating inflammatory cells, leading to airway smooth muscles contract, triggering asthma. Through network pharmacology analysis, it is found that *β*-sitosterol can bind to key targets on the pathways related to asthma response [21]. Chan et al. found through research that the *PTGS2* gene is closely related to the inflammation of the asthmatic response, and the cyclooxygenase encoded by the *PTGS2* gene has an airway remodeling effect [22]. Kaempferol can slow down the asthma response by breaking down fat or regulating fatty acid metabolism [23]. From the molecular mechanism of our result, it is found that PRA has a strong inhibitory effect on the inflammation of the respiratory system. Further studies are warranted to clarify the role of PRA in the treatment of asthma via the TNF-*α* signaling pathway.

In summary, this study used network pharmacology to analyze the main active ingredients of PRA. The main active ingredients and their possible molecular mechanisms for the treatment of asthma have been clarified. These results provide a theoretical basis of PRA for the treatment of asthma and provide a scientific basis for its better clinical application.

## Figures and Tables

**Table 1:** Candidate active ingredients of PRA.

Mol ID	Molecule name	OB (%)	DL
MOL001902	3*β*,23-Dihydroxy-oleana-11,13(18)-dien-28-oic acid	21.53	0.75
MOL001910	11Alpha,12alpha-epoxy-3beta-23-dihydroxy-30-norolean-20-en-28,12beta-olide	64.77	0.38
MOL001911	Albiflorin R1	21.29	0.82
MOL001912	Albiflorin R1_qt	26.18	0.34
MOL001918	Paeoniflorgenone	87.59	0.37
MOL001919	(3S,5R,8R,9R,10S,14S)-3,17-Dihydroxy-4,4,8,10,14-pentamethyl-2,3,5,6,7,9-hexahydro-1H-cyclopenta[a]phenanthrene-15,16-dione	43.56	0.53
MOL001921	Lactiflorin	49.12	0.8
MOL001924	Paeoniflorin	53.87	0.79
MOL001925	Paeoniflorin_qt	68.18	0.4
MOL001928	Albiflorin_qt	66.64	0.33
MOL001929	Alexandrin	20.63	0.62
MOL001930	Benzoyl paeoniflorin	31.27	0.75
MOL001933	Oxypaeoniflorin	21.88	0.78
MOL000211	Mairin	55.38	0.78
MOL000263	Oleanolic acid	29.02	0.76
MOL000358	Beta-sitosterol	36.91	0.75
MOL000359	Sitosterol	36.91	0.75
MOL000422	Kaempferol	41.88	0.24
MOL000492	(+)-Catechin	54.83	0.24
MOL000551	Hederagenol	22.42	0.74
MOL000357	Sitogluside	20.63	0.62

**Table 2:** Target-gene pairs of active ingredients of PRA.

Target gene	Gene name
26S proteasome non-ATPase regulatory subunit 3	PSMD3
5-Hydroxytryptamine 2A receptor	HTR2A HTR2
5-Hydroxytryptamine receptor 3A	HTR3A
Acetylcholinesterase	ACHE
Activator of 90 kDa heat shock protein ATPase homolog 1	AHSA1
Aldo-keto reductase family 1 member C3	AKR1C3
Alpha-1D adrenergic receptor	ADRA1B
Androgen receptor	AR
Antileukoproteinase	**SLPI**
Apoptosis regulator BAX	BAX
Apoptosis regulator Bcl-2	BCL2
Arachidonate 5-lipoxygenase	ALOX5
Aryl hydrocarbon receptor	AHR
Beta-2 adrenergic receptor	ADRB2 ADRB2R, B2AR
Beta-lactamase	blaC
Calmodulin	CAMKK2
Caspase-3	CASP3
Caspase-8	CASP8
Caspase-9	CASP9 MCH6
Catalase	CAT
Cell division control protein 2 homolog	CDC42
CGMP-inhibited 3′,5′-cyclic phosphodiesterase A	PDE3A
Coagulation factor VII	F7
Coagulation factor Xa	F10
Cytochrome P450 1A1	CYP1A1
Cytochrome P450 1A2	CYP1A2
Cytochrome P450 1B1	CYP1B1
Cytochrome P450 3A4	CYP3A4
Cytochrome P450-cam	camC
Dipeptidyl peptidase IV	DPP4
DNA topoisomerase II	TOP2
Dopamine D1 receptor	DRD5 DRD1B, DRD1L2
E-Selectin	SELE
Estrogen receptor	ESR1
Gamma-aminobutyric acid receptor subunit alpha-1	GABRA1
Gamma-aminobutyric-acid receptor alpha-2 subunit	GABRA2
Gamma-aminobutyric-acid receptor alpha-3 subunit	GABRA3
Gamma-aminobutyric-acid receptor alpha-5 subunit	GABRA5
Glutathione S-transferase Mu 1	GSTM1
Glutathione S-transferase Mu 2	GSTM2
Glutathione S-transferase P	GSTP1
Heat shock protein HSP 90	HSP90AA1 HSP90A, HSPC1, HSPCA
Heme oxygenase 1	HMOX1 HO, HO1
Hyaluronan synthase 2	HAS2
Inhibitor of nuclear factor kappa-B kinase subunit beta	IKBKB
Insulin receptor	INSR
Intercellular adhesion molecule 1	ICAM1
Interleukin-6	IL6
Interstitial collagenase	MMP1
Lipopolysaccharide-binding protein	LBP
Microtubule-associated protein 2	Map2
Mineralocorticoid receptor	NR3C2
Mitogen-activated protein kinase 8	MAPK8
Monocyte differentiation antigen CD14	CD14
mRNA of PKA catalytic subunit C-alpha	PRKACA
Muscarinic acetylcholine receptor M1	CHRM1
Muscarinic acetylcholine receptor M2	CHRM2
Muscarinic acetylcholine receptor M3	CHRM3
Muscarinic acetylcholine receptor M4	CHRM4
Mu-type opioid receptor	OPRM1 MOR1
NAD(P)H dehydrogenase [quinone] 1	NQO1 DIA4, NMOR1
Neuronal acetylcholine receptor protein, alpha-7 chain	CHRNA7 NACHRA7
Neuronal acetylcholine receptor subunit alpha-2	CHRNA2
Nitric-oxide synthase, inducible	NOS2
Nitric-oxide synthase, endothelial	NOS3
Nuclear receptor coactivator 2	**NCOA2**
Nuclear receptor subfamily 1 group I member 2	NR1I2
Pancreatic alpha-amylase	AMY2A
Peroxidase C1A	PRXC1A
Peroxisome proliferator activated receptor gamma	PPARG
Phosphatidylinositol-4,5-bisphosphate 3-kinase catalytic subunit, gamma isoform	PIK3CG
Potassium voltage-gated channel subfamily H member 2	KCNH2 ERG, ERG1, HERG
Progesterone receptor	PGR
Prostaglandin G/H synthase 1	PTGS1
Prostaglandin G/H synthase 2	PTGS2
Protein kinase C-alpha type	PRKCA
RAC-alpha serine/threonine-protein kinase	AKT1
Retinoic acid receptor RXR-alpha	RXRA
Serine/threonine-protein phosphatase 2B catalytic subunit alpha isoform	PPP3CA
Serum paraoxonase/arylesterase 1	PON1
Signal transducer and activator of transcription 1-alpha/beta	STAT1
Sodium channel protein type 5 subunit alpha	SCN5A
Sodium-dependent noradrenaline transporter	SLC6A2
Solute carrier family 2, facilitated glucose transporter member 4	SLC2A4
Thrombin	F2
Transcription factor AP-1	JUN
Transcription factor p65	RELA
Transforming growth factor beta-1	TGFB1
Trypsin-1	PRSS1
Tumor necrosis factor	TNF

**Table 3:** Degree of top 5 chemicals and target proteins of PRA.

Gene	Degree	Molecule	Degree
PGR	6	Kaempferol	63
NCOA2	5	Beta-sitosterol	39
HSP90	4	Sitogluside	17
PTGS1	4	(+)-Catechin	11
PTGS2	4	Oleanolic acid	6

**Table 4:** Common target genes of PRA and asthma.

Comment target genes
Arachidonate 5-lipoxygenase (5-LOX)
DNA topoisomerase II (TOP2)
E-selectin (SELE)
Muscarinic acetylcholine receptor M2 (CHRM2)
Muscarinic acetylcholine receptor M3 (CHRM3)
Muscarinic acetylcholine receptor M4 (CHRM4)
Neuronal acetylcholine receptor alpha-7 (CHRNA7)
Nitric-oxide synthase inducible (NOS2)
Prostaglandin G/H synthase (COX/PTGS1)
Prostaglandin G/H synthase 2 (COX-2/PTGS2)
Tumor necrosis factor (TNF)
Heat shock protein 90 (HSP90)

**Table 5:** KEGG pathway enrichment analysis of key 13 genes (part).

Term	Input	*P* value
P00003:Alzheimer disease-amyloid secretase pathway	4	5.76*E* − 09
hsa04725:cholinergic synapse	4	5.33*E* − 08
hsa04080:neuroactive ligand-receptor interaction	5	6.40*E* − 08
hsa00590:arachidonic acid metabolism	3	1.33*E* − 06
hsa04668:TNF signaling pathway	3	7.12*E* − 06
hsa04726:serotonergic synapse	3	7.69*E* − 06
hsa05310:asthma	1	0.0107
H00079:asthma	1	0.00368

## Data Availability

Source data of this study is derived from the public repositories, as indicated in “Methods” of the manuscript. All data that support the findings of this study is available from the corresponding authors upon reasonable request.
